# Implications of ABCG2 Expression on Irinotecan Treatment of Colorectal Cancer Patients: A Review

**DOI:** 10.3390/ijms18091926

**Published:** 2017-09-07

**Authors:** Dorte Lisbet Nielsen, Jesper Andreas Palshof, Nils Brünner, Jan Stenvang, Birgitte Martine Viuff

**Affiliations:** 1 Department of Oncology, Herlev and Gentofte Hospital, University of Copenhagen, DK-2730 Herlev, Denmark; dorte.nielsen.01@regionh.dk (D.L.N.); jesper.andreas.palshof@regionh.dk (J.A.P.); 2 Section of Molecular Disease Biology, Department of Drug Design and Pharmacology, Faculty of Health and Medical Sciences, University of Copenhagen, DK-2100 Copenhagen, Denmark; nbr@sund.ku.dk (N.B.); bmv842@gmail.com (B.M.V.)

**Keywords:** ABCG2, BCRP, colorectal cancer, irinotecan

## Abstract

Background: One of the main chemotherapeutic drugs used on a routine basis in patients with metastatic colorectal cancer ((m)CRC) is the topoisomerase-1 inhibitor, irinotecan. However, its usefulness is limited by the pre-existing or inevitable development of resistance. The ATP-binding cassette (ABC) transporter ABCG2/breast cancer resistance protein (BRCP) through its function in xenobiotic clearance might play an important role in irinotecan resistance. With a goal to evaluate the clinical significance of ABCG2 measurements, we here review the current literature on ABCG2 in relation to irinotecan treatment in CRC patients. Results: Few studies have evaluated the association between *ABCG2* gene or protein expression and prognosis in CRC patients. Discordant results were reported. The discrepancies might be explained by the use of different criteria for interpretation of results in the immunohistochemistry studies. Only one large study evaluated the ABCG2 protein expression and efficacy of irinotecan in mCRC (CAIRO study, *n* = 566). This study failed to demonstrate any correlation between ABCG2 protein expression in the primary tumor and response to irinotecan-based treatment. We recently raised questions on how to evaluate ABCG2 immunoreactivity patterns, and the results in the CAIRO study might be influenced by using a different scoring protocol than the one proposed by us. In contrast, our recent exploratory study of *ABCG2* mRNA expression in 580 patients with stage III primary CRC (subgroup from the randomized PETACC-3 study) indicated that high *ABCG2* tumor tissue mRNA expression might be predictive for lack of efficacy of irinotecan. Conclusion: The biological role of ABCG2 in predicting clinical irinotecan sensitivity/resistance in CRC is uncertain. In particular, the significance of ABCG2 cellular localization needs to be established. Data concerning *ABCG2* mRNA expression and prediction of adjuvant irinotecan efficacy are still sparse and need to be confirmed.

## 1. Introduction

Colorectal cancer (CRC) has a high incidence and mortality worldwide. In 2012, CRC was the second most prevalent cancer type in men (9%) and the third among women (8%) [1]. Approximately 15–25% of patients with CRC present with synchronous liver metastases. In addition, nearly 50% of patients initially diagnosed with localized disease ultimately develop metastases [2]. Randomized clinical CRC trials have established the superiority of 5-fluorouracil (5-FU) in combination with oxaliplatin or irinotecan in metastatic (m)CRC [3]. Moreover, a number of biologically active substances targeting specific signaling pathways in cancer cells have been approved in the treatment of patients with CRC, e.g., cetuximab, panitumumab, and bevacizumab. Owing to these treatments, the overall survival of mCRC patients has been significantly improved. However, initial or acquired tumor drug resistance is still a common observation among mCRC patients, resulting in a high treatment failure leading to a high mortality rate among these patients.

Irinotecan (CPT-11) is a derivative of camptothecin and its active metabolite is SN-38, which is an inhibitor of topoisomerase 1, a nuclear enzyme needed for the replication and transcription of supercoiled DNA [4,5]. Several cellular mechanisms of resistance to irinotecan have been reported. Among these are reduced intracellular drug accumulation mediated by the ATP binding cassette (ABC) efflux transporter ABCG2 encoded by the *ABCG2* gene, also known as breast cancer resistance protein (BRCP), the placental ABC transporter or mitoxantrone resistance-associated protein [6].

The human ABC proteins belong to one of the largest superfamily of transporters and are divided into seven subfamilies, ABCA to ABCG, which comprise a total of 48 members (http://www.genenames.org/genefamilies/ABC). Among these members, the three efflux transporters ABCB1 (*P*-glycoprotein), ABCC1 (the multidrug resistance protein 1 (MRP1)), and ABCG2 are recognized as major contributors to multidrug resistance in human cancer cells [7].

ABCG2 processes a very broad substrate specificity that differs from, but substantially overlaps with that of ABCB1 and ABCC1 [8]. More recently, ABCG2 has been recognized by the US Food and Drug Administration (FDA) to be one of the key drug transporters involved in drug absorption and elimination. We have recently shown that acquired resistance to SN-38 in human CRC and breast cancer cell lines results in a major upregulation of the *ABCG2* transcript and protein, and that the specific inhibition of ABCG2 results in the re-sensitization of the resistant cells [9,10], which strongly suggests a key role of this protein in SN-38 resistance.

With this review, we aim to determine the significance of ABCG2 measurement in predicting clinical resistance to irinotecan in CRC patients.

## 2. Method

PubMed was searched independently by two authors (DN and JAP) using the following search strategy: ABCG2 AND irinotecan; BCRP AND irinotecan; ABCG2 AND “colorectal cancer” OR “colorectal neoplasms”; BCRP AND “colorectal cancer” OR “colorectal neoplasms” (four searches). A total of 243 publications were identified. Abstracts from the annual meetings of the American Society of Clinical Oncology (ASCO) were retrieved for relevant abstracts using the same search terms. Articles fulfilling the following criteria were excluded: reviews, duplications, non-human studies, pre-clinical studies, studies in other cancer types, studies not involving the *ABCG2* gene or protein, studies on the toxicity or efficacy of other drugs, and studies not published in English. The last search was carried out in July 2017. Ultimately, 13 studies were included in this review.

## 3. ABCG2

*ABCG2* was initially cloned from multidrug-resistant breast cancer cell lines and demonstrated to confer resistance to chemotherapeutic agents such as mitoxantrone, topotecan, and SN-38 [11,12,13]. Since then, the number of substrates has been rapidly expanding to include other chemotherapeutic drugs such as methotrexate and several tyrosine kinase inhibitors (TKI) (imatinib and gefitinib). Notably, several drugs which are integral parts of the current treatment of CRC such as irinotecan and 5-FU are substrates of ABCG2 [14].

Physiological substrates include estrone-3-sulfate, 17β-estradiol 17-(β-d-glucuronide), and uric acid. Additionally, a range of common dietary xenobiotics are also substrates [7]. Hundreds of inhibitors with diverse chemical structures have been identified, including calcium channel blockers and drugs like tamoxifen and omeprazole [15,16,17].

The cloning of *ABCG2* cDNA from drug-selected cell clones and normal tissue has demonstrated functional variations in the amino acid substitutions in the protein with unaltered substrate specificity (for a comprehensive review, see Noguchi et al.) [18].

In normal tissue, the ABCG2 protein is highly expressed in the apical membrane of the placental syncytiotrophoblasts, the epithelium of the small intestine, colon, and rectum, and on the biliary canalicular membrane of hepatocytes. Furthermore, the protein is expressed on the luminal membrane of brain microvessel endothelial cells and, to lesser extent, on kidney proximal tubular cells. The tissue localization suggests a crucial role in absorption, distribution, metabolism, and elimination (ADME) of endogenous substances and xenobiotics. Today, it is clear that the protein plays a key role in ADME of anticancer drugs [17].

The transporter is frequently expressed on malignant hematopoietic and lymphoid cells, and evolving literature associates *ABCG2* single nucleotide polymorphisms (SNPs) not only with anti-cancer drug efficacy, but also with incidence of leukaemia [19]. ABCG2 is also known as a stem cell marker in immature myeloid and leukaemia stem cells [20]. Interestingly, subpopulations of stem-like cells expressing ABCG2 (side population) have been found in a wide range of solid tumors, including head and neck cancer, breast-, lung-, ovarian-, pancreatic- and CRC [21,22]. These stem-like cells have characteristics of stem cells, e.g., quiescence, drug resistance, enhanced self-renewal capacity, and tumorigenicity. The role of ABCG2 in drug resistance in this subpopulation of cells is currently under investigation [23]. In CRC, Wnt signaling has been found to expand this side population in SW480 cells in vitro. These side population cells expressed both *ABCG2* and *ABCB1* and displayed resistance to 5-FU and SN-38 [24]. Finally, tumor cells isolated from 13 surgically resected colon tumors and propagated as solid tumor spheroids in vitro have been shown to possess stem cell properties including self-renewal, expression of *ABCG2*, and resistance to SN-38 [25].

## 4. *ABCG2* Gene and Protein Expression in CRC; Findings Concerning Prognosis and Prediction

Studies concerning prognosis are given in Table 1. Recently, a cancer stem cell gene profile including *AGCG2* was found to predict relapse in 62 patients with stage II and III, radically resected CRC receiving adjuvant chemotherapy with either single agent 5-FU (or capecitabine) or 5-FU (or capecitabine) in combination with oxaliplatin [26], as patients (19%) with an unfavorable cancer stem cell profile had a relapse-free survival of 22 months versus 43 months for patients (81%) with a favorable profile.

Candeil et al. [27] investigated the expression of mRNA in normal colon tissue (eight samples), primary CRC tumor tissue (10 samples), and CRC hepatic metastases (constituting 23 untreated, 12 after irinotecan-based therapy, and four after non-irinotecan chemotherapy). The tumors did not seem to be matched. The study showed a significant decrease in the expression of *ABCG2* mRNA in the primary tumor tissue (about 100-fold compared with normal tissue), and about a five-fold increase in *ABCG2* mRNA expression in the metastases from irinotecan-treated patients. However, the expression was still significantly lower than in normal colon mucosa cells (about 15-fold). This data can be interpreted as primary colon cancer cells exhibiting an initial downregulation of *ABCG2* mRNA expression, suggesting that the downregulation of *ABCG2* might be involved in the carcinogenesis of CRC and, moreover, and that some primary CRC patients will benefit from adjuvant irinotecan treatment (see below). Moreover, a change in *ABCG2* expression may occur during the progression of CRC (from primary to metastatic disease, which might be related to the systemic treatment applied).

Furthermore, a study of normal tissue and matched primary tumors from 13 CRC patients showed that the *ABCG2* mRNA was decreased six-fold in cancer tissue compared to matched normal CRC mucosa. No significant correlation between *ABCG2* mRNA expression and age, sex, race, grade or stage of tumors, location, or treatment efficacy was established [28]. Finally, a study of *ABCG2* mRNA showed no significant difference in the expression in 21 patients with mucinous and 30 patients with non-mucinous primary CRC, respectively. The number of patients with recurrent disease (17) was too limited to perform subgroup analyses for patients receiving, e.g., irinotecan [29].

We recently published a paper on the analytical validation of anti-ABCG2 antibodies for immunohistochemistry (IHC) on formalin-fixed paraffin-embedded tumor tissue [33]. This study clearly showed that not all commercially available anti-ABCG2 antibodies are suited for IHC. Moreover, based on the obtained results, we proposed a scoring protocol for ABCG2 immunoreactivity in CRC.

A Chinese study of ABCG2 protein expression analyzed with IHC (IHC; sparse information about antibodies: multiclonal anti-ABCG2 antibody; a FOUR score system based on the intensity of immunostaining and the number of positive cells was used, no separate scores for cytoplasmic versus membrane immunostaining) and including 60 cases of primary CRC showed that ABCG2 positive cells were mainly positioned in the front of the carcinomatous tissue (the invasion front) or between carcinomatous and non-carcinomatous margin tissue. ABCG2 was expressed in 36.7% of carcinomatous tissue as compared to 3.3% in non-carcinomatous margin tissue (cell type not specified) (*p* < 0.001). Furthermore, a high protein expression of ABCG2 was found in 30% of cases with lymph node metastases compared to 6.7% in cases without positive lymph nodes (*p* < 0.025). The protein was mainly expressed in the membranes of the cells. The results prompted the authors to suggest that ABCG2 might be important in the progression of CRC [30].

A study by Wang et al. [31] investigated ABCG2 in 225 primary CRC tissues using IHC (antibody mouse monoclonal antibody, BXP-21, Abcam Company, Cambridge, MA, USA; a FOUR score system based on the intensity of immunostaining and the number of positive cells was used with separate scores for cytoplasmic and membrane immunostaining). Totally, 83.1% of the cases showed positive cytoplasmic expression, including 13.3% which were strongly positive, whereas 66.2% showed positive membranous expression including 15.6% strongly positive. The strong membranous staining significantly correlated to higher Dukes’ stage, more lymph nodes, and the presence of distant metastases. Furthermore, membranous staining strongly correlated to a shortened survival (Hazard Ratio (HR) 2.44, 95% Confidence Interval (CI) 1.05–5.65; *p* = 0.038) [31]. The cytoplasmic expression levels were only correlated by increasing Dukes’ stage. Of note, clinical follow-up data was only available for 69 patients (39.1%).

More recently, a Korean study by Kang et al. [32] examined 234 consecutive patients who underwent surgical resection of primary CRC. ABCG2 expression was evaluated by IHC (antibody: rabbit polyclonal antibody, Santa Cruz Biotechnology, Dallas, TX, USA; a scoring system based on the intensity of immunostaining and the number of positive cells was used; the composite score was dichotomized at the median with separate scores for cytoplasmic and membrane immunostaining) and staining in the cytoplasm and membrane was more frequent in well-differentiated lesions and was observed in 78% and 62% of the tumors, respectively. In contrast to the abovementioned study by Wang, the study by Kang showed that high membranous expression of ABCG2 was significantly associated with better overall survival (OS) (HR 0.62; 95% CI 0.41–0.95; *p* = 0.027) and disease-specific survival (HR 0.50; 95% CI 0.31–0.81; *p* = 0.005), while cytoplasmic expression of ABCG2 was not significantly associated with survival.

Only four studies investigated the ability of ABCG2 to predict sensitivity to irinotecan (Table 2) [9,34,35,36]. Recently, we published an exploratory study of *ABCG2* mRNA expression (dichotomized by medium value) in 580 evaluable patients with colon cancer stage III enrolled in the adjuvant PETACC-3 prospective randomized study [9,37]. This study enrolled 3278 patients and was designed to evaluate the efficacy of the addition of adjuvant irinotecan to standard 5-FU/leucovorin. The results strongly suggested a predictive role of tumor *ABCG2* mRNA expression. The median *ABCG2* expression was used to split patients into “high” or “low” *ABCG2* groups. The survival curves showed a trend towards a significant separation in the irinotecan receiving patients, with *ABCG2* levels below the median being associated with a longer overall survival. However, such an association was not observed in the patients treated with 5-FU only.

A first-line study of 5-FU and irinotecan (FOLFIRI) in 58 patients with mCRC investigated the association between ABCG2 protein expression determined by IHC (antibody: a mouse monoclonal antibody, BXP-21, Abcam Company, Cambridge, MA, USA; a FOUR score system based on the intensity of immunostaining and the number of positive cells was used with no separate score for membrane versus cytoplasmic immunostaining) and clinical outcome. Furthermore, associations between thymidylate synthase (TS), topoisomerase 1 (TOP 1), carboxylesterase (CES2) protein expression, and clinical outcome were investigated. ABCG2 cytoplasmic expression was found in 56% of the patients, with most positive tumor samples showing a membranous staining and some diffuse cytoplasmic staining. No correlations with clinicopathological characteristics and ABCG2 expression (grading 0 + 1 versus 2 + 3) were demonstrated. Moreover, the expression of the protein did not affect response rate (RR) (determined according to RECIST 1.1 [38]), time to progression (TTP), or OS. In addition, ABCG2 protein expression was investigated in 19 synchronous liver metastases and 17 metastatic lymph nodes, and no correlation between ABCG2 expression and primary tumors and metastatic lesions was observed. Additional analyses revealed that only TS significantly correlated with clinical outcome (RR, TTP, and OS) [34].

Trumpi et al. [35] investigated ABCG2 and ABCB1 protein expression in 566 patients with mCRC included in the CAIRO study [39]. In this study, the authors analyzed tumor samples from patients who received either first-line treatment with capecitabine, second-line irinotecan, and third-line capecitabine plus oxaliplatin (sequential treatment; *n* = 410) or first-line treatment with capecitabine plus irinotecan and second-line capecitabine plus oxaliplatin (combination treatment; *n* = 410). ABCG2 protein expression was determined by IHC (ABCG2 antibody: a mouse monoclonal antibody, BXP-21, Abcam Company, Cambridge, MA, USA; using a scoring system based on luminal membrane and cytoplasmic expression, tissue microarray (TMA) with one core from each patient). The authors found no significant difference with regard to response (determined according to RECIST 1.0 [40]) after the first cycle of chemotherapy between ABGG2-positive and -negative tumors to either monotherapy (*p* = 0.879) or combination therapy (*p* = 0.102) with irinotecan. In addition, a multivariate analysis showed that ABCG2 expression was neither an independent predictor for progression-free survival (PFS) in patients treated with irinotecan monotherapy (5.7 versus 6.1 months; *p* = 0.811) nor in patients treated with capecitabine and irinotecan (9.0 versus 10.0 months; *p* = 0.196).

Finally, 189 patients who underwent colorectal resection were enrolled in a retrospective study of ABCG2 protein expression determined by IHC (ABCG2 antibody: a mouse monoclonal antibody, BXP-21, Abcam Company, Cambridge, MA, USA). Seventeen patients received irinotecan-based chemotherapy for recurrent disease. In a multivariate logistic regression analysis, increased expression of ABCG2 was an independent predictor of resistance to SN-38 (response evaluation was performed according to RECIST 1.1 [38]). Furthermore, patients with increased levels of ABCG2 had shorter PFS than patients with low levels (104 versus 242 days; *p* = 0.047) [36].

## 5. Studies on Inhibitors of ABCG2 in CRC

To date, many ABCG2 inhibitors with diverse chemical structures have been identified and the list is continually growing [16]. Among these, Ko143, a highly potent analogue of fumetremorgin C1, has been shown to re-sensitize human cancer cell lines with acquired SN-38 resistance and significant upregulation of *ABCG2* mRNA as well as to enhance the efficacy of irinotecan in ABCG2-expressing CRC xenograft tumor models [41,42]. Yet, no clinical studies with Ko143 were identified. Although most TKIs are competitive or high affinity substrates of ABCG2, some of them such as sorafenib, sunitinib, lapatinib, erlotinib, gefitinib, imatinib, and nilotinib have been reported to be ABCG2 inhibitors or modulators [43,44,45,46,47]. These TKIs seem to inhibit the function of ABCG2 by directly interacting with the substrate-binding sites, thus acting as competitive antagonists [48]. More recently, afatinib has been shown to significantly inhibit the ATPase activity of ABCG2 and to downregulate the mRNA and protein expression of the transporter [49]. Some of these TKIs appear also to be involved in resistance mediated by ABCB1 (e.g., imatinib and nilotinib) [50,51].

Mazard et al. used both in vitro and in vivo CRC models (nude mouse xenografts) to investigate sorafenib in combination with SN-38 or irinotecan. Sorafenib did inhibit ABCG2-mediated transport and enhanced intracellular drug accumulation, and thus increased the efficacy of irinotecan [54]. However, the authors concluded that the inhibition of ABCG2 was clearly not the only mechanism involved in the synergy between sorafenib and irinotecan. Interestingly, sorafenib has also been shown to improve the efficacy of irinotecan by inducing ABCG2 degradation in lysosomes and by the inhibition of irinotecan-mediated p38 and extracellular signal-regulator kinase (ERK) activation [44,55].

Studies of inhibitors of ABCG2 are given in Table 3. A phase I, dose escalation study of sorafenib in combination with irinotecan included 20 patients with refractory solid tumors in cohorts 1–3 (previous systemic therapy 90% of patients, previous topoisomerase 1 directed therapy not reported) and 14 patients with mCRC in cohort 4 (prior irinotecan not reported) [52]. Stable disease was reported in 60% of evaluable patients with solid tumors, and 77% of patients in the extended part of the study (duration nor reported). One patient with mCRC had a partial response. However, prior to the study, this patient had only received 5-FU and oxaliplatin. Sorafenib 100 or 200 mg BID had no impact on the pharmacokinetics of irinotecan or its metabolite SN-38 as measured in plasma, whereas sorafenib 400 mg two times a day significantly increased the area under the concentration curve (AUC) of irinotecan and SN-38. This was not associated with increased toxicity. The authors suggested that the increase of SN-38 concentration in the plasma was due to inhibition by sorafinib of the formation of SN-38 glucuronide, since sorafenib strongly inhibited SN-38 glucuronidation in human liver microsomes in vitro. No analysis of tumor tissue ABCG2 expression was performed [52].

NEXIRI was a phase I/II trial of sorafenib plus irinotecan as second- or later-line of treatment in patients with *KRAS*-mutated mCRC. The phase II part of the trial included 54 mCRC patients who had all progressed after irinotecan-based chemotherapy. Efficacy data were promising, with a disease control rate (complete response + partial response + stable disease (duration not reported)) of 64.9% (95% CI 51–77%), a median PFS of 3.7 months (95% CI 3.2–4.7 months), and a median survival (OS) of 8.0 months (95% CI 4.8–9.7 months). Toxicities included 37% grade 3 diarrhea, 13% grade 3 hand-foot syndrome, and 18% and 17% grade 3 and 4 neutropenia, respectively [53]. No pharmacokinetic analysis of irinotecan was performed. Furthermore, the expression of ABCG2 was not determined. Thus, the mechanism involved was not elucidated and further investigations are needed. A randomized phase II study of irinotecan, sorafenib, and the combination of the two drugs is ongoing (NEXIRI 2; ClinicalTrials.gov NCT01715441). In addition, two other phase II studies have investigated the combination of FOLFIRI and sorafenib (NCT00839111) and sorafenib with FOLFOX or FOLFIRI (NCT00889343). No data have yet been published.

## 6. Discussion

Overall, a very limited number of studies describing the association between *ABCG2* or ABCG2 protein expression and irinotecan efficacy in CRC patients have been published. Two studies compared the expression of *ABCG2* mRNA in normal colon tissue and tumor tissue. Both studies showed decreased expression in tumor tissue [27,28]. This finding prompted Gupta et al. to suggest that *ACCG2* could play a role in tumorigenesis by allowing the accumulation of genotoxins and the overproduction of nitric oxide. However, both studies were small (13 and 62 patients) and the results need to be confirmed in larger series.

Few studies evaluated the association between *ABCG2* gene or protein expression and prognosis in CRC patients. It is of note that different criteria for the interpretation of results in these IHC studies were used (see below). One study found that high expression of stem cell markers including ABCG2 correlated with poor patient prognosis [26], and another study found high expression of ABCG2 to be correlated to the presence of lymph node metastases [30]. Two studies performed separate assessment of membrane and cytoplasmic expression of ABCG2. One of these studies found that high membranous expression of ABCG2 correlated with shorter OS [31]. In contrast, a recent study found high membranous expression of the protein to be significantly associated with better OS [32]. A small study including only 10 primary tumors, 22 untreated hepatic metastases, and 12 hepatic metastases obtained after irinotecan-containing chemotherapy showed increased *ABCG2* mRNA in hepatic metastases after exposure to irinotecan, suggesting a role of *ABCG2* in the development of acquired irinotecan resistance in vivo [27]. More recently, we published data on *ABCG2* mRNA expression and benefit (DFS and OS) in stage III colon cancer patients receiving an adjuvant combination of 5-FU or 5-FU plus irinotecan [9]. This study suggested a predictive role of tumor *ABCG2* mRNA expression since a separation of the survival curves by the median *ABCG2* mRNA expression in the irinotecan receiving patients was observed (patients with high *ABCG2* mRNA expression had a worse outcome), while such a separation was not observed in the 5-FU-only treated patients. Since no difference was observed in the 5-FU-only treated patients, this study thus supported the lack of a prognostic role of ABCG2. More importantly, the separation of the survival curves among the patients receiving irinotecan pointed to a predictive utility of *ABCG2* mRNA measurement in the adjuvant irinotecan treatment of patients with colon cancer.

In contrast, a study evaluating the expression of ABCG2 protein and the efficacy of irinotecan-based treatment in 58 patients with mCRC failed to demonstrate any correlation between ABCG2 and outcome [34], and a study of ABCG2 and ABCB1 protein expression in 566 patients from the prospective CAIRO study did not show any association between expression of the proteins and response to irinotecan [35]. Furthermore, in the last study, neither ABCG2 nor ABCB1 were independent predictors of PFS.

Overall, the role of *ABCG2* as a prognostic factor or as predictor for irinotecan efficacy in CRC is not well established. The few studies reported seemed to report discordant results.

Several explanations can be proposed for this lack of consistency. First of all, some studies measured the *ABCG2* mRNA expression [9], whereas other measured the ABCG2 protein expression by applying the BXP21 antibody in IHC in either whole sections [34,36] or TMAs [35]. Moreover, the study by Silvestris et al. [34] was not powered to detect any differences between protein expression and drug effect. In the study by Trumpi et al., all included patients received irinotecan [35]. Only one study complied with the recommendations put forward by Simon et al. [56] on the use of archived biopsies for evaluating prognostic and predictive biomarkers. Thus, only the study by Jensen et al. [9] included a control group not receiving irinotecan, allowing for distinction between prognostic and predictive markers. Secondly, no validated assays and standardized reference values for ABCG2 IHC exist today. Obviously, this leads to difficulties when comparing results, as most studies have used individual cut-offs and varying assays to assess the protein expression. Furthermore, different locations of ABCG2 (basolateral/luminal versus cytoplasmic) may fundamentally influence the results. For many other IHC tests, e.g., HER2, validated scoring protocols have been implemented. However, for ABCG2 IHC, no such standard scoring protocols have yet been presented, although such uniform guidelines are a prerequisite for comparing results from different clinical studies. In addition, tumor heterogeneity might influence the results as some of the studies used TMAs. In analogy to P-glycoprotein (ABCB1), it is likely that the different methods, including issues such as pre-analytical, analytical, post-analytical variations, reproducibility, and sensitivity, that have been used to evaluate the expression levels and the functional activity of ABCG2 may greatly contribute to the observed discrepancies in the monitoring of ABCG2 expression [57,58,59]. Finally, it should also be noted that ABCG2 expression monitored by mRNA or protein level may have limited reliability with respect to protein function and thus to the cellular drug resistance [57].

All clinical studies but one focused on one transporter protein at a time, whereas it might be that tumor cells depend on several drug transporters at the same time, executing their function in concert. On the other hand, investigating both ABCG2 and ABCB1, Trumpi et al. did not find any correlation between each of the ABC transporters or the combination of the two transporters and outcome [35]. Also, the protocol used for fixation, as well as the timing of biopsies differ from one study to another, resulting in different exposures to chemotherapeutics which could influence the gene and protein expressions. In particular, Trumpi et al. investigated a patient cohort in which irinotecan was used as the second-line therapy [35]. First-line (and adjuvant) therapy may have influenced the expression level of ABCG2. Indeed, preclinical research has shown that 5-FU in combination with oxaliplatin may alter the expression of these proteins [60]. Of note, Trumpi et al. evaluated the concordance of ABCG2/ABCB1 expression in 17 primary tumors and their corresponding metastases and found a mediocre to poor concordance. Finally, in vitro, it has been shown that ABCB1-mediated resistance depends on transporter expression levels [61,62]. Most studies have found the expression of drug transporters in clinical samples to be significantly lower than in drug-selected cell lines [63,64]. Thus, the fact that the cancer cells express an increased level of the transporter does not automatically mean that the transporter mediates significant resistance to the applied chemotherapy.

Currently, only one clinical phase I/II study aiming to overcome drug resistance by inhibiting ABCG2 by sorafinib has been reported [53]. The phase II part of the trial included 54 mCRC patients who were exposed to sorafinib plus irinotecan. All of the patients had previously failed irinotecan treatment. Efficacy data were promising with a disease control rate of 65%, a median PFS of 3.7 months, and a median OS of 8.0 months. A randomized phase II is currently ongoing (NEXIRI 2; ClinicalTrials.gov NCT01715441). Unfortunately, no test for the expression of *ABCG2* prior to inclusion is included and the role of *ABCG2* might therefore not be elucidated.

In general, however, studies on inhibitors of ABC transporters have been disappointing [65]. In addition, the co-administration of ABCG2 inhibitors might increase the toxicity of irinotecan in tissues which are protected by the physiological expression of ABCG2/ABC transporters. Notably, it has recently been demonstrated that the inhibition efficiency of competitive inhibitors of ABCB1 depend on the expression level of ABCB1. Thus, cells expressing high levels of the transporter require higher concentrations of competitive inhibitors to achieve the same reversal of the drug efflux [66]. These findings might also be applicable for ABCG2. When introducing novel ABCG2 inhibitors into clinical testing, these drugs will be administered together with chemotherapy, e.g., irinotecan. The potential increase in irinotecan toxicity in normal cells expressing ABCG2 efflux pumps (e.g., hepatocytes, brain-blood barrier cells), will always be of great concern. We presently have a novel ABCG2 inhibitor in clinical trials (SCO-101) and, having successfully passed four clinical phase 1 studies as monotherapy, we are now preparing a clinical phase II study where the initial part will be a run-in study consisting of a limited dose escalation study with increasing doses of SCO-101 and a standard dose of chemotherapy [67]. Another important piece of information regarding normal tissue toxicity when bypassing ABCG2 concerns studies where cancer patients have received novel types of topoisomerase inhibitors that are not substrates for ABCG2 [68,69,70]. These phase 1 studies did not show unexpected toxicities in normal tissue and thus support the idea of combining ABCG2 inhibitors with standard chemotherapy. These findings might be also applicable for ABCG2.

ABCB1 and ABCG2 are known to act as components of the blood-brain barrier [71] and thus, if effective inhibitors could be identified, might lead to increased drug uptake in the brain. Although brain metastases are relatively infrequent in patients with gastrointestinal cancers, CRC patients with *RAS* mutations have an increased incidence of brain metastases [72,73]. Finally, during the recent years, evidence has mounted to indicate that many cancers including CRC display subpopulations of stem cells that are responsible for tumor self-renewal. Such stem cells are characterized by the expression of *ABCG2* as well as other ABC transporters. Of specific interest is the inhibition of ABCG2 as means of sensitizing cancer stem cells to chemotherapy, which is currently under investigation [17].

In conclusion, only a few studies have investigated the role of ABCG2 in CRC. The limited data suggest that ABCG2 tumor cell expression might not be associated with patient prognosis. Two large studies, one analyzing *ABCG2* mRNA and adjuvant irinotecan treatment, the other investigating the predictive value of ABCG2 protein/mRNA expression in mCRC patients receiving irinotecan treatment, have shown contradictory results. A phase I/II study suggests that a TKI might increase the efficacy of irinotecan, and the results of a subsequent randomized phase II are eagerly expected. Based on this review, we conclude that the biological role of ABCG2 in clinical drug resistance is still unknown, as is the clinical value of determining *ABCG2* mRNA expression, and/or ABCG2 protein expression in relation to prognosis and drug prediction. However, before such studies are undertaken, we suggest that international efforts be made to standardize ABCG2 measurements.

## Figures and Tables

**Table 1:** Studies of *ABCG2* gene and protein expression in CRC in relation to expression pattern and/or prognosis.

Reference	Setting	Treatment	Number of Patients	Methods	“Biomarker” Investigated	Findings	Conclusions; Comments
Giampieri, et al. [26]	Primary stage II and III	5-FU/capecitabine ± oxaliplatin	62	Quantitative PCR	Panel of 66 genes for “stemness” including *ABCG2*	“Unfavorable cancer stem cell profile” (19%): Relapse-free survival: 22 months Favorable profile (81%): Relapse-free survival 43 months	Expression levels of cancer stem cells genes may be relevant for the prognosis; *ABCG2* among genes with high “weight”
Candeil, et al. [27]	Normal colon tissue Primary tumour Hepatic metastases	Untreated Untreated Irinotecan-based therapy Chemotherapy without irinotecan	8 10 23 12 4 non-matched	Semi-quantitative RT-PCR	*ABCG2* mRNA	Highly expressed 1/100 of normal colon tissue Five-fold compared with primary tumor NS from primary tumor	*ABCG2* mRNA expression might be upregulated by irinotecan treatment, suggesting the potential involvement of *ABCG2* in irinotecan resistance
Gupta, et al. [28]	Normal colon tissue Primary tumour	-	13 (matched)	Semi-quantitative RT-PCR	*ABCG2* mRNA	6.6 ± 0.6-fold lower in cancer compared to controls (*p* < 0.0001) No correlation with grade or stage of tumor, or with treatment	*ACCG2* mRNA may have a role in tumorigenesis, allowing the accumulation of genotoxins and the overproduction of nitric oxide
Glasgow, et al. [29]	Primary, Dukes’ stage C	-	21 mucinous 30 nonmucinous	RT-PCR	*ABCG2* mRNA	No difference in mucinous and nonmucinous tumors	17 with recurrent disease; subset analysis of patients receiving irinotecan not possible
Liu, et al. [30]	Primary	-	60	IHC; whole sections; Multiclonal antibody	ABCG2	36.7% of carcinomatous tissue; mainly membranous expression Correlation to lymph node metastases	ABCG2 may be important in the progression and metastasis of CRC
Wang, et al. [31]	Primary	-	225	IHC; whole sections; A mouse monoclonal antibody, BXP-21	ABCG2	83% positive cytoplasmic expression, 13% high 66% positive membranous expression, 16% high High membranous expression correlated to shorter OS Cytoplasmic expression not associated with OS	Membranous ABCG2 expression is a potential independent prognostic factor
Kang, et al. [32]	Primary	88.5% received 5-FU-based adjuvant chemotherapy	234	IHC; TMA; a rabbit polyclonal antibody	ABCG2	78% positive cytoplasmic expression 62% positive membrane High membranous expression associated with better OS Cytoplasmic expression not associated with OS	Membranous ABCG2 expression is a potential prognostic factor

5-FU: 5 Fluorouracil; CRC: colorectal cancer; IHC: immunohistochemistry; NS: not significant; PCR: polymerase chain reaction; RT-PCR: reverse transcription polymerase chain reaction; OS: overall survival; TMA: tissue microarray.

**Table 2:** Studies of *ABCG2* gene and protein expression in CRC in relation to the prediction of outcome after irinotecan-based therapy.

Reference	Setting	Treatment	Number of Patients	Methods	“Biomarker” Investigated	Findings	Conclusions; Comments
Jensen, et al. [9]	Primary stage II and III	Randomized phase III; 5-FU vs. 5-FU + irinotecan	688; statistical analysis performed on 580 stage III	Microarray gene expression analysis	*ABCG2* mRNA	A separation of the survival curves by the median *ABCG2* mRNA expression in the irinotecan receiving patients was observed, while such a separation was not observed in the 5-FU-only treated patients	A predictive role of tumour *ABCG2* mRNA expression is strongly suggested
Silvestris, et al. [34]	Metastatic, 1st line	FOLFIRI	58	IHC; whole section; mouse monoclonal antibody BXP-21	ABCG2	56% high expression; no association to clinicopathological characteristics; no correlation to RR, TTP, OS	No predictive role for ABCG2 protein expression was found
Trumpi, et al. [35]	Metastatic 1st or 2nd line	Capecitabine, irinotecan (sequential or combination; CAIRO study)	566	IHC; TMA, mouse monoclonal antibody BXP-21	ABCG2	Response to irinotecan was not significantly different in tumors with positive vs negative expression of ABCG2. ABCG2 was not an independent predictor of PFS	ABCG2 protein does not predict response to irinotecan
Tuy, et al. [36]	Metastatic, 1st line	Irinotecan-based regimens Other regimens (not specified)	17 171	IHC; whole section; mouse monoclonal antibody BXP-21	ABCG2	Tumors with increased expression of ABCG2 were significantly more resistant to irinotecan	Increased expression of ABCG2 is an independent predictor of SN-38 resistance (risk of resistance increased 12-fold)

CRC: colorectal cancer; FOLFIRI: irinotecan + 5-FU + leucovorin; IHC: immunohistochemistry; PFS: progression-free survival; RR: response rate, OS: overall survival; TMA: tissue microarray; TTP: time to progression; vs: versus.

**Table 3:** Studies on inhibitors of ABCG2 in CRC.

Reference	Treatment	Phase	Number of Patients	Population	Status	DCR	Median PFS (95%CI) (Months)	Median OS (95%CI) (Months)
Mross, et al. [52]	Irinotecan + sorafenib	I	20 + 14	Various solid tumours, mCRC	Completed	60% 85%	NR	NR
Samalin, et al. [53]	Irinotecan + sorafenib (NEXIRI)	I/II	10 + 54	*KRAS* mutated mCRC; 2nd or later lines (67% ≥ 3 prior lines)	Completed	Phase I: 78 Phase II: 64.9 (51–77)	Phase II: 3.7 (3.2–4.7)	Phase II: 8.0 (4.8–9.7)
NCT01715441	Irinotecan or sorafenib Irinotecan + sorafenib (NEXIRI 2)	II, randomized	160 planned	*KRAS* mutated mCRC, failure of all known drugs	Ongoing	Estimated study completion: September 2015; no published data		
NCT00839111	FOLFIRI + sorafinib	II	43 planned	mCRC, failure of oxaliplatin-based therapy	Ongoing, not verified since September 2010	Estimated study completion: November 2010; no published data		
NCT00889343	Irinotecan/oxaliplatin + sorafenib Irinotecan/oxaliplatin + placebo	II, randomized	101 planned	mCRC, 2nd line	Completed	No published data		

DCR: disease control rate; mCRC: metastatic colorectal cancer; NEXIRI: combined sorafenib and irinotecan; NR: not reported; PFS: progression-free survival; OS: overall survival.
